# Targeting the Polyadenylation Signal of Pre-mRNA: A New Gene Silencing Approach for Facioscapulohumeral Dystrophy

**DOI:** 10.3390/ijms19051347

**Published:** 2018-05-03

**Authors:** Anne-Charlotte Marsollier, Romain Joubert, Virginie Mariot, Julie Dumonceaux

**Affiliations:** 1 NIHR Biomedical Research Centre, University College London, Great Ormond Street Institute of Child Health and Great Ormond Street Hospital NHS Trust, London WC1N 1EH, UK; annecharlotte.marsollier@gmail.com (A.-C.M.); r.joubert@ucl.ac.uk (R.J.); virginie.mariot@ucl.ac.uk (V.M.); 2 Laboratoire Reproduction et Développement des plantes, Université de Lyon, ENS de Lyon, UCB Lyon 1, CNRS, INRA, F-69342 Lyon, France

**Keywords:** polyadenylation, alternative polyadenylation, muscular diseases, facioscapulohumeral dystrophy, myotonic dystrophy, therapeutic strategies

## Abstract

Facioscapulohumeral dystrophy (FSHD) is characterized by the contraction of the D4Z4 array located in the sub-telomeric region of the chromosome 4, leading to the aberrant expression of the DUX4 transcription factor and the mis-regulation of hundreds of genes. Several therapeutic strategies have been proposed among which the possibility to target the polyadenylation signal to silence the causative gene of the disease. Indeed, defects in mRNA polyadenylation leads to an alteration of the transcription termination, a disruption of mRNA transport from the nucleus to the cytoplasm decreasing the mRNA stability and translation efficiency. This review discusses the polyadenylation mechanisms, why alternative polyadenylation impacts gene expression, and how targeting polyadenylation signal may be a potential therapeutic approach for FSHD.

## 1. Introduction

Several molecules targeting RNAs have been developed during the last few years to treat patients affected by muscle diseases, including RNA interference and synthetic antisense oligonucleotides (AON). These molecules can (i) prevent formation of the mRNA 5′cap; (ii) modulate RNA splicing by masking keys sequences; (iii) influence the use of an alternative polyadenylation site; (iv) induce a RNAse H1-dependent degradation of the mRNA, and (v) inhibit the mRNA translation via steric blockade of ribosome access to mRNA (for review see [1]). In this review, another possibility is investigated: targeting the polyadenylation signals to destabilize the pre-mRNA.

## 2. Polyadenylation Mechanisms

The vast majority of eukaryotic mRNAs, with the exception of replication dependent histone mRNAs, present a similar 3′end characterized by a long chain of adenine nucleotides called the poly(A) tail. This poly(A) tail, which is not encoded by the DNA, is formed after endonucleolytic cleavage of the primary transcript followed by the addition of adenine nucleotides at the 3′OH end of the cleavage product. The specificity and efficiency of this 3′end processing are determined by the binding of a multiprotein complex to regulatory cis-acting sequence elements on the pre-mRNA. 

### 2.1. Cis-Regulatory Sequence Elements

**Figure 1:**
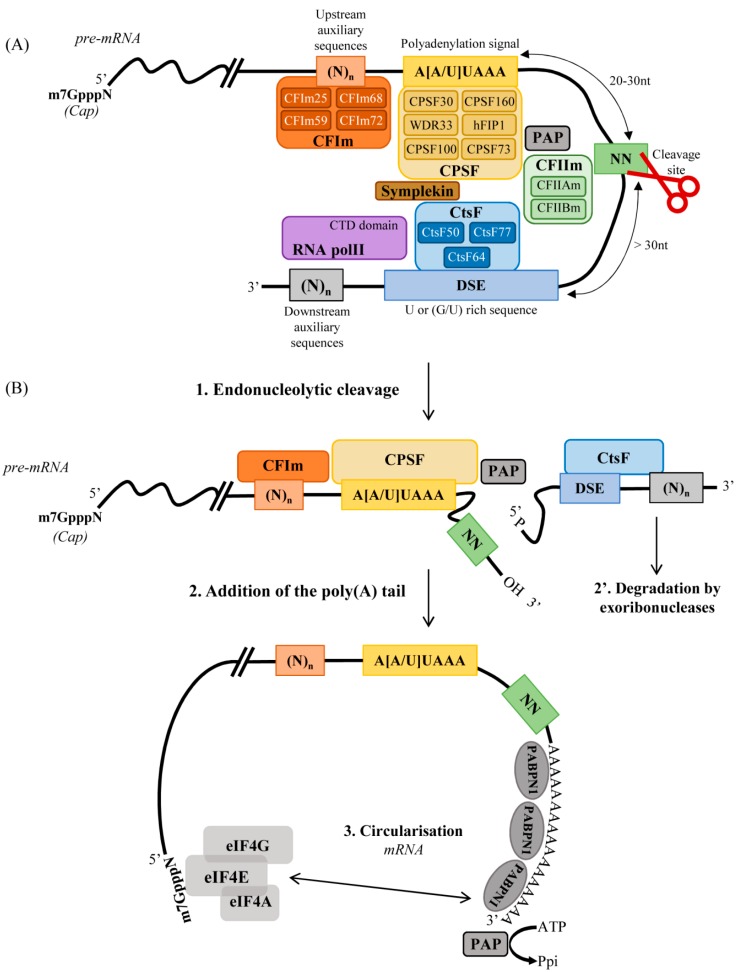
*Cis*-regulatory sequence elements and protein factors involved in cleavage and polyadenylation. (**A**). The specificity and efficiency of 3′end processing is determined by the binding of more than 80 RNA-binding proteins to regulatory *cis*-acting RNA sequence elements including: the polyadenylation signal (PAS) A[A/U]UAAA; the cleavage site (represented by NN) and the downstream sequence element (DSE). Auxiliary sequences can be found near the polyadenylation signal or the DSE. The core processing complex, which is sufficient for the cleavage and polyadenylation, is composed of approximatively 20 proteins, distributed in 8 complexes: the cleavage and polyadenylation specificity factor (CPSF), the cleavage stimulation factor (CstF); the mammalian cleavage factors I (CFIm) and the mammalian cleavage factors II (CFIIm); the single protein poly(A) polymerase (PAP); the single protein poly(A)-binding protein nuclear 1 (PABPN1); the single protein RNA polymerase II large subunit (Pol II); and the symplekin. Subunits of the different factors are indicated. (**B**). CPSF and CstF are co-transcriptionally recruited to the poly(A) signal and the DSE respectively, causing an endonucleolytic cleavage of the pre-mRNA between the PAS and the DSE at the cleavage site. Two fragments are generated: one fragment with a free 5′phosphate group which is rapidly degraded by exoribonucleases and one fragment with a free 3′hydroxyl group on which 250 adenines will be added by PAP. The newly synthetized poly(A) tail is covered by PAPBN1, allowing mRNA circularization and stabilization.

Few regulatory sequences on the pre-mRNA play a major role in the 3′end processing. The most important sequence element is probably the polyadenylation (poly(A)) signal (PAS), defined by an hexameric consensus sequence (usually A[A/U]UAAA) located ~20–30 nucleotides upstream of the cleavage site (Figure 1A). This hexamer is not strictly conserved, and several variants have been identified [2,3,4] (Table 1). These variants are not as efficient as the canonical one and the analysis of their distribution suggests they may be used for regulatory purposes (Table 1) [2,3,4,5]. Polymorphisms in PAS are rare, highlighting the importance of the sequence conservation in evolution [6]: alterations in the PAS have been frequently associated with diseases, such as β-thalassemia (OMIM #613985) or immunodysregulation polyendocrinopathy enteropathy X-linked syndrome (IPEX, OMIM #304790) (For review see [7]).

The second regulatory sequence is the downstream sequence element (DSE), characterized by a high density of uracil (U) and/or guanine/uracil (G/U) residues and located more than 30 nucleotides downstream of the cleavage site [8,9,10] (Figure 1). There is no clear consensus sequence for the DSE, but its nucleotide composition seems to impact the 3′end processing efficiency [11,12].

A third cis-regulatory sequence is the cleavage site, located between 20 and 30 nucleotides downstream of the PAS (Figure 1) [4,13]. The poly(A) tail attachment begins at this cleavage site (frequently at a CA or a UA dinucleotide in mammals). The nucleotide composition around the cleavage site is heterogeneous and importantly, impacts the cleavage efficiency [14,15].

Finally, additional auxiliary sequences, located upstream or downstream of the cleavage site, can also influence the 3′end processing efficiency: (i) U-rich auxiliary sequences (USE) [3,9]; (ii) G-rich auxiliary sequence elements leading to the formation of G-quadruplex structures [9,16,17]; (iii) AUA auxiliary element for a selected set of mRNAs polyadenylated by the non-canonical poly(A) polymerase Star-PAP [15,18,19,20,21]; or (iv) distal auxiliary elements located downstream from the PAS [22].

### 2.2. Core Processing Complex

The cleavage and polyadenylation reactions are governed by more than 80 RNA-binding proteins but less than 20 factors compose the core of the processing complex and are necessary and sufficient to mediate cleavage and polyadenylation in vitro [23,24]. These 20 factors are distributed in eight complexes (Figure 1) [23,25]:

- Cleavage and polyadenylation specificity factor (CPSF): is a multiprotein complex implicated in the PAS recognition and the cleavage of the pre-mRNA [26,27,28]. The core of CPSF complex is composed of CPSF100 and CPSF73 which form a heterodimer and recruit the other CPSF subunits and symplekin [29,30]. CPSF73 has a zinc-dependent endonuclease activity that is essential for the pre-mRNA cleavage. It has a very weak enzymatic activity suggesting that other factors may be required for an efficient cleavage [31,32]. The specific interaction of CPSF with the hexameric poly(A) signal is mediated by WDR33, CPSF30, and CPSF160, while hFip1 binds the U-rich sequences [26,33,34,35,36]. Finally, hFip1 and CPSF160 recruit by direct interaction the poly(A) polymerase (PAP) to the PAS [26,37].
- Cleavage stimulation factor (CstF): is essential for the cleavage reaction but not for the polyadenylation reaction [38,39]. CstF is an multimeric protein complex made up of subunits CstF64, CstF77, and CstF50 [28,40] which are respectively involved in (i) the specific recognition of the DSE region (by CstF64), (ii) the assembly of the CstF complex (by CstF77), (iii) the CstF-CPSF interaction (strong interaction between CPSF160 and CstF77), (iv) the interaction with the C-terminal domain of the RNA polymerase PolII (PolII) (by CstF50), and (v) the interaction with the breast cancer 1 (BRCA1) associated really interesting new gene (RING) domain 1 (BARD1) complex (by CstF50) to inhibit the pre-mRNA 3′end processing during DNA repair/following DNA damage, reducing errors in the mRNA [41,42,43,44].
- Symplekin: is considered to be a scaffolding protein connecting CPSF and CstF and supporting the assembly of the polyadenylation machinery [27,28,45].
- Mammalian cleavage factor I (CFIm): influences alternative poly(A) site selection, mRNA transport and mRNA splicing [45,46,47,48,49,50]. It is a heterodimer composed of the smallest CFIm25 subunits and any of the largest CFIm68, CFIm59, or CFIm72 subunits [51,52,53,54]. CFIm binds UGUA motifs, typically located upstream of the PAS [9,55,56].
- Mammalian cleavage factor II (CFIIm): is required only for the cleavage step [57,58,59] and is composed of two subunits: CFIIAm, which is required for the cleavage reaction; and CFIIBm, which acts as a stimulator for the cleavage [23].
- Poly(A) polymerase (PAP): catalyzes the reaction leading to the addition of 200–250 adenosines as polyadenosine tail to the newly synthesized pre-mRNA molecules [60,61,62]. PAP is recruited by CPSF [26] and its activity is stimulated by the poly(A) binding protein nuclear 1 (PABPN1), which plays a major role in poly(A) tail length control [63,64,65]: the binding of PABPN1 to the newly synthesized polyadenosine tail accelerates the rate of adenosine addition mediated by PAP [62,63,64]. PABPN1 covers the entire length of the poly(A) tail during and after the polyadenylation reaction. When the poly(A) tail reaches 200–250 adenosines, the polyadenylation reaction is stopped [63]. The length of the poly(A) tail appears to be critical for a suitable gene expression: transcripts with short or long poly(A) tails are retained in the nucleus and degraded [66,67,68].

The RNA polymerase II (PolII) plays a critical role by coupling pre-mRNA processing to transcription [69,70]. The PolII C-Terminal Domain (CTD) may promote the assembly of a 3’-end processing complex through an interaction with CPSF, CstF, and CFIIm [71,72,73]. PolII may also be required for efficient pre-mRNA cleavage [71].

Besides these factors, other proteins participating in the 5′ end capping can also influence the cleavage and polyadenylation efficiency [74,75,76].

### 2.3. Polyadenylation Steps

The first step of the 3′end processing is the co-transcriptional recruitment of CPSF and CstF to the PAS and the DSE respectively [77,78]. CPSF and CstF are loaded onto PolII during elongation [69,70,79] and after the transcription of the PAS, PolII pauses allowing the binding of CPSF to the hexameric sequence through WDR33 and CPSF30 [70]. CstF then interacts with the U/GU rich sequences in the DSE via CstF64 [80] and once the CPSF and CstF components are linked, additional factors—including CFIm and CFIIm—join the complex around the future cleavage region [77]. The assembly of all of these proteins induces an endonucleolytic cleavage of the pre-mRNA between the PAS and the DSE, generally 20–30 nucleotide downstream of the hexameric sequence (Figure 1B). The cleavage efficiency can be influenced by 5′ cap structure such as the nuclear cap-binding complex [75]. Two fragments are generated: one with a free 3′hydroxyl group and the other one with a free 5′phosphate group which is immediately degraded by the Xrn2 exoribonuclease (Figure 1) [81,82]. In parallel, PAP is anchored to the cleaved pre-mRNA by an interaction with CPSF and starts adding adenines [26,37]. The newly synthetized poly(A) tail is covered by PABPN1 proteins which control its length [62,63,64,65]. When the polyadenylation process is complete, the 3′end processing complex is disassembled. PAPBN1 thus interacts with the initiation factor proteins located in the 5′ untranslated region (UTR) leading to the mRNA circularization and stabilization [58,59,77,83,84]. The mature mRNA is then ready to be exported to the cytoplasm. 

## 3. Alternative Polyadenylation

### 3.1. General Regulation of Alternative Polyadenylations

In mammals, 70–80% of transcripts have at least two alternative PAS leading to cleavage at different sites and production of different mRNA transcripts—a phenomenon called alternative polyadenylation (APA) [85,86]. A multitude of factors is involved in the selection of the 3′-end processing site. We will only focus on the most important and those described in muscle cells. Among these factors is the PAS hexameric sequence itself, which is not identical in the distal and proximal PAS sites. Most distal sites tend to use the canonical A[A/U]UAAA sequence whereas the proximal sites tend to use variant signals [2]. Most 3′-APA also tends to be associated with stronger and more conserved *cis*-regulatory sequence elements (upstream U-rich elements and DSE) [42]. Since the variant PAS signals are processed less efficiently than the canonical ones, the use of a proximal PAS may be done for regulatory purposes. However, it is still far from clear how a particular PAS is chosen and many factors may influence this choice. Alternative polyadenylation has been described to be regulated by many factors, including the cell proliferation and differentiation state, the distance between two PAS, the PolII polymerase speed, the presence of a pause site downstream of the proximal PAS, the presence of methylated CpG islands, nucleosome occupancy or histone methylation, and the concentration of the different proteins involved in the polyadenylation steps [2,87,88,89,90,91,92,93,94,95,96,97,98,99]. Splicing and polyadenylation are also interconnected and are likely to be in competition with each other in case of an intronic PAS. For example, ablation of the splicing factor 3B subunit1 (a component of U2 snRNP) activates intronic PAS [97]. U1 snRNP also affects cleavage and polyadenylation independently of its role in splicing. The use of cryptic PAS within introns close to the 5′-end of the transcript is increased in the case of knockdown of U1 snRNP, potentially because U1 snRNP binding to these regions blocks their recognition by cleavage factors [100,101]. 

### 3.2. Alternative Polyadenylations and Their Consequences

**Figure 2:**
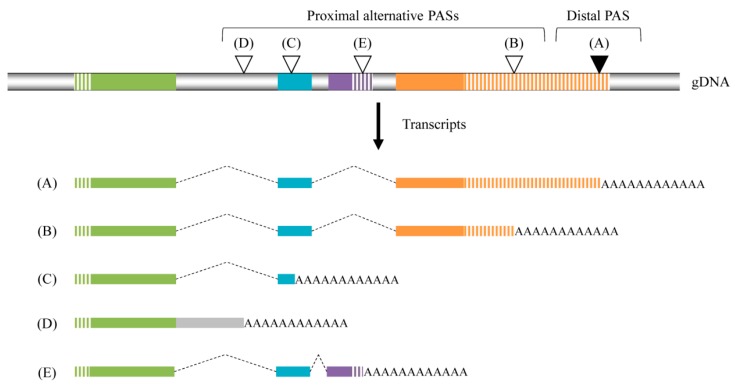
Alternative polyadenylations (APA). APAs have several patterns. The use of (**A**) a constitutive polyadenylation at a distal site leads to the normal mRNA and protein. (**B**) A proximal site located in a non-coding sequence of the mRNA results in 3′UTR shortening without any modification of the protein. (**C**) An APA located in a proximal exon (or in the coding sequence of the last exon) leads to truncated proteins. (**D**) An APA located in an intron (i.e., involving a cryptic poly(A) site in introns) and leads to a modified protein with an alternative C-terminus or to truncated proteins (depending of the presence of a stop codon). (**E**) An APA located in an alternative terminal exon APA, due to the use of an alternative splicing, leads to a protein with a different C-terminus. Introns are in grey or represented by a dotted line when spliced; non-coding sequence are hatched. Distal PAS is represented by a black arrowhead and proximal alternative PASs by a white one.

Several patterns define the APAs. The most frequent is the presence of multiple PAS in the 3′UTR of the terminal exon. Cleavage and polyadenylation at any of these sites will lead to transcript isoforms that differ in the length of the 3′UTR, with an identical protein-coding region (Figure 2A,B). Therefore, 3′UTR APA is more likely to affect post-transcriptional gene regulation through the modulation of mRNA stability, translation, nuclear export and cellular localization [86]. Although two recent articles have demonstrated that 3′UTR shortening may have a limited effect on mRNA stability [102,103], isoforms with long 3′UTRs are believed to be less stable than short isoforms because they can bind more destabilizing elements. Global analyses have revealed that APA influences around 10% of predicted targets between any two cell types analyzed [104]. The length of the 3′UTR also influences the mRNA subcellular localization and long 3′UTR isoforms are also more abundant in the nucleus [105].

Another pattern of APA is defined by the presence of a cryptic PAS in internal exons or in introns (Figure 2C,D). Transcripts produced from an internal exon or intronic APA cannot possess an in-frame stop codon or 3′UTR and are likely to be degraded rapidly through the non-stop mediated mRNA decay [106]. In the absence of a stop codon, the poly(A) tail could also generate a C-terminal poly-lysine tag which is generally unstable [107,108]. When present in the coding sequence, APAs can lead to the expression of truncated proteins with new functions as seen in the retinoblastoma binding protein 6 (OMIM #600938) [109,110,111].

A last form of APA, involving alternative exons, leads to proteins lacking domains or with alternative sequences (Figure 2E). For example, the immunoglobulin M (IgM) heavy chain has two transcripts, resulting in the shift from a distal PAS to an internal site and then the substitution of the two terminal exons, coding the amino acid sequence for membrane-binding, by a sequence involved in the secretion [112,113].

### 3.3. Alternative Polyadenylations in the Skeletal Muscle World

A growing number of studies about APAs has been published during recent years, in which APAs are associated with knockdown, depletion, or overexpression of different proteins or genes. Here, we will focus only on APAs connected to the “skeletal muscle world”.

APAs have been described to play a role in muscle metabolism and myogenesis. For example, (i) slow and fast muscle fibres display 7% of their transcriptome with different APAs [114]. (ii) *PAX3* and *PAX7*, coding for two transcription factors involved in myogenesis, are also subject to APAs resulting in resistance to miR-206 regulation or alternative C-terminal domain [115,116]. (iii) During myogenesis, differential expression, and localization of copper transporters are associated with mRNA 3′UTR shortening of the transporter *ATP7A* [117]. (iv) *UCP3* has an intronic APA leading to a protein without an inhibition site at the C-terminus [118]. This may play a role in the pathogenesis of dystrophies because UCP3 is involved in the mitochondrial proton leak and the limitation of reactive oxygen species production [119], and consequently in oxidative stress regulation. (v) One of the most recognized major players in controlling muscle mass is mammalian target of rapamycin (mTOR) whose activation increases protein synthesis and prevents atrophy (for review see [120]). One of the molecular signatures of mTOR activation includes 3′UTR shortening of mRNAs leading to the overexpression of selected E2 and E3 components in ubiquitin ligase complexes resulting in elevated levels of protein ubiquitination [121]. This phenomenon could be required for the continuous supply of amino acids to cellular systems, to maintain the steady-state protein synthesis [122]. (vi) Like mTOR, the androgen receptor (AR) is a well-known regulator of muscle anabolism (for review see [123]). Some prostate cancers are castration-resistant due to *AR* splice variants that are constitutively active transcription factors. These variants lack the ligand-binding domain thanks to the use of an APA in a cryptic exon [124]. 

PAS can also be blocked by protein and/or RNA elements that compete with the 3′-end processing machinery. For example, mutations in PABPN1 causes oculopharyngeal muscular dystrophy (OPMD, OMIM #164300), characterized by progressive degeneration of muscles in adults [125]. Mutated PABPN1 aggregates in the nucleus and forms filamentous nuclear inclusions. While this protein is not involved in the choice of PAS, its knockdown produced a shorter 3′UTR [126,127], suggesting that PABPN1 could act prior to cleavage and polyadenylation of pre-mRNA to determine the PAS used. It was proposed that PABPN1 competes with CPSF for binding to A-rich regions at proximal, consensual PAS inhibiting its usage. When PABPN1 is depleted or mutated (in OMPD), CPSF can recognize the previously hidden PAS. Interestingly, whereas PABPN1 is ubiquitously expressed and presumably contributes to control of gene expression in all tissues, mutation of the PABPN1 gene only affects a limited set of skeletal muscles, most likely because PABPN1 levels are dramatically lowered in skeletal muscle compared to other tissues [128], thus highlighting the importance of the concentration of the different proteins involved in the polyadenylation steps in APA.

APA patterns may also be involved in the onset of myotonic dystrophy (DM1, OMIM #160900). Indeed, DM is characterized by the re-emergence of developmentally immature alternative splicings (AS) and APA patterns in adult tissues because proteins of the MBLN family are titrated, leading to immature AS. Gene ontology and systems analysis reveals several different classes of misregulated genes in APA, including those involved in ubiquitination, IGF-1 signalling, and the mTOR pathway [129].

## 4. Therapeutic Strategies Targeting Polyadenylation in Muscle Diseases

Because polyadenylation is essential for gene expression, strategies aiming at disrupting gene expression by targeting the polyadenylation have been developed. These strategies might be particularly important for gain of function diseases such as DM1 or facioscuplohumaral dystrophy (FSHD, OMIM #158900) which are two of the three most prevalent muscle diseases with an estimated prevalence of 4/100,000 and 4.5/100,000, respectively. 

**Figure 3:**
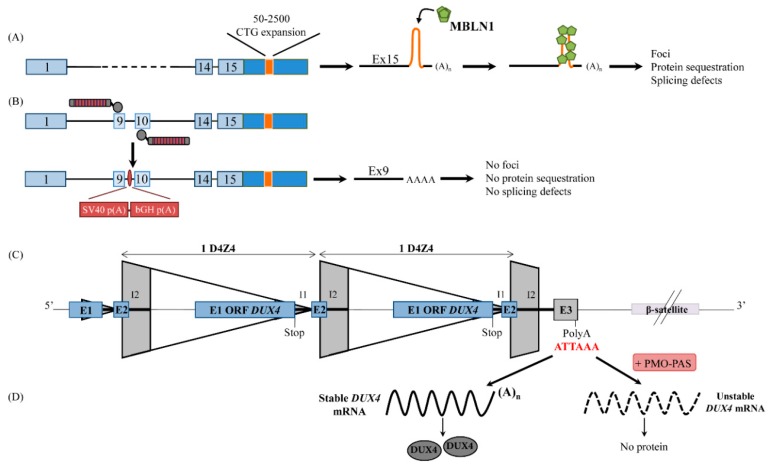
Therapeutic strategies targeting polyadenylation in muscle diseases. (**A**) In Myotonic dystrophic type 1 patients, the mutated DPMK gene carries between 50 to 2500 CTG expansions in the 3′UTR leading to the formation of a stem-loop which sequesters splicing factors such as MBLN1, causing foci formation and splicing defects. (**B**) Two PAS (SV40p(A) and bGH p(A)) have been inserted between exons 9 and 10 (by the TALEN system), allowing the elimination of the mutant transcript. The presence of a stop codon located at the beginning of intron 9 leads to a truncated mRNA *DPMK* (Figure 2D) which no longer carries the toxic CUG repeats [135]. (**C**) *DUX4* ORF is located in each D4Z4 repeat but the polyadenylation signal is in exon 3 (E3) positioned in the sub-telomeric part of the chromosome 4. The hypomethylation of the D4Z4 region, when associated with a permissive chromosome 4, leads to the aberrant expression of the DUX4 transcription factor and the mis-regulation of hundreds of DUX4 target genes. (**D**) In the presence of the PMO-PAS targeting the DUX4 PAS, correct polyadenylation of DUX4 is inhibited, leading to an unstable *DUX4* mRNA which is not translated [136,137].

DM1 is a multisystemic disease and patients show an extremely widely variable phenotype. The symptoms include myotonia, muscle wasting, cardiac conduction defects, cataracts, and insulin resistance (for review see [130]). DM1 is an inherited monogenic disorder characterized by a repeat expansion in the *Dystrophia Myotonica Protein Kinase* (*DMPK*) gene localized on chromosome 19. The 3′UTR of this gene normally contains 5–37 copies of a CTG trinucleotide repeat while the most severely affected DM1 patients harbor between 50 and several thousand repeats [131]. The mutation is thought to adopt a stem-loop structure within the mRNA [132] and numerous mechanisms have been proposed to explain how CUG-expansion in the 3′UTR untranslated region of an mRNA creates such adverse multisystemic effects including the aberrant alternative splicing of several key mRNAs, the alterations in the usage of alternative polyadenylation sites of a number of mRNAs and diffusion of the molecular pathological phenotype through nuclear protein spreading (for review see [133]) [134] (Figure 3A). DM1 thus appears to be the result of a highly stable hairpin mRNA structure in the *DPMK* mRNA which facilitates binding/sequestration of several factors, mainly leading to the misregulation of several splicing events and dysregulation of translation.

FSHD pathology is characterized by an atrophy of the muscles of the face, shoulders, and arms, leading to muscle weakness and asymmetric involvement of affected musculature [138,139]. In 95% of FSHD patients (named FSHD1, OMIM #158900), a contraction of the D4Z4 array, located in the sub-telomeric region of chromosome 4, is observed [140]. This contraction is associated with a loss of repressive epigenetic marks within the D4Z4 macrosatellite, leading to the expression of the *DUX4* gene which is composed of three exons. The DUX4 ORF is fully included in the first exon, whereas exons 2 and 3 are non-coding regions (3′UTR). Importantly, exon 3 is located outside of the D4Z4 repeats and carries the *DUX4* PAS (Figure 3C) [141,142]. Two allelic variants (4qA and 4qB) exist in this region distal to D4Z4 but FSHD is only associated with the 4qA variant [143] which contains a functional but non-canonical PAS (AUUAAA) [141]. The remaining 5% of FSHD patients (named FSHD2, OMIM #158901) do not show the D4Z4 contraction but carry a mutation in the epigenetic modifier genes *SMCHD1* or *DNMT3B* [144,145], also leading to the hypomethylation of the D4Z4 array and to the aberrant expression of the DUX4 protein in the context of a permissive chromosome 4. Even if other genes may participate in the onset of FSHD [146,147] DUX4 is believed to play a major role in disease onset and/or progression. Indeed, (i) DUX4 protein and mRNA are detected in both adult and fetal FSHD1 and FSHD2 muscle cells and biopsies [148,149,150], (ii) hypomethylation of the D4Z4 array is always observed in FSHD patients, (iii) individuals carrying a permissive chromosome 4 but lacking the hypomethylation of the D4Z4 array are asymptomatic carriers [151], and (iv) the expression of DUX4 accounts for the majority of the gene expression changes in FSHD skeletal muscles [152].

Both FSHD and DM1 are gain-of-function diseases and so far, there is no curative or preventive treatment for these pathologies. Several strategies have been proposed in the literature, and in this review we will focus on those altering the polyadenylation signal. Mutated *DMPK* and *DUX4,* being the causative genes of DM1 and FSHD respectively, were targeted by strategies using either a TALEN-based or an antisense oligonucleotide approach [135,136,137] (Figure 3B,D). In myotonic dystrophy, the authors used an approach that they had previously developed on neural stem cells, which was the insertion of two poly(A) signals upstream of *DMPK* CTG repeats in intron 9, thus leading to premature cleavage of transcript before the transcription of the toxic region [153]. In this case, the introduced PASs may have created an APA which inhibits intron 9 splicing and an in frame stop codon located at the beginning of intron 9 is used (Figure 2D). The authors used DM1 induced pluripotent stem (iPS) cells and demonstrated that integration of a PAS upstream of the CTG repeats eliminated nuclear RNA foci in the treated cells, even after their differentiation into neural cells or cardiomyocytes. Aberrant splicings were also abolished. Interestingly, when the PAS was inserted between the *DMPK* stop codon and the start of the CTG repeats, none of the clones had the mutant allele targeted, potentially because of heterochromatin spreading caused by the expanded CTG repeats. 

In FSHD, a systematic analysis of the cis-acting elements that govern *DUX4* cleavage and polyadenylation has been performed and revealed that sequences downstream of the SNP located within the β-satellite region are critical for *DUX4* cleavage and polyadenylation [22]. Antisense oligonucleotides targeting the mRNA of a GFP reporter construct carrying these distal auxiliary elements led to a decrease in GFP expression, thus suggesting that these elements could have therapeutic potential [22]. 

Two independent groups have targeted the *DUX4* PAS using antisense phosphorodiamidate morpholino oligonucleotides (PMO) in FSHD cells [136,137]. Different 3′key elements of the *DUX4* mRNA were targeted by several PMOs and the same PMO (PMO-PAS) was found to be the one giving the best extinction of *DUX4* mRNA in both studies (Figure 3D). This PMO precisely targets the PAS which is in a region of an open conformation, whereas the cleavage site and the DSE lay within a closed region. In FSHD myotubes, *DUX4* downregulation in the presence of PMO-PAS is associated with a downregulation of many transcriptional targets of DUX4 without particular off-target effects. In vivo, after electroporation of PMOs into FSHD patient muscle xenografts in immunodeficient mice, DUX4 target genes are also downregulated [136]. Remarkably, whereas other PAS are present in the subtelomeric region of chromosome 4, downstream from the pathological one, none of them seems to be used in the presence of the PMO [137]. However, one of the PMOs used in the Marsollier et al. publication (PMO-CS3) induces a switch in cleavage site usage ~40 nt upstream of the normal one [137], thus suggesting that an unidentified alternative PAS was used. The use of this APA allows *DUX4* mRNA to escape, at least partially, the degradation process which occurs in non-polyadenylated mRNA. Moreover, because this new cleavage site is located upstream of the normal one, it is possible the truncated *DUX4* mRNA may be more stable. The mechanisms leading to the use of an upstream APA are not known and need to be deciphered.

## 5. Conclusions

During the past 20 years, many laboratories have used antisense oligonucleotides to silence gene expression. Targeting the 3′end element of mRNA is a new approach that offers several advantages: (i) all polyadenylated mRNAs may be targeted using this strategy since polyadenylation is a crucial and common step required for the maturation of all eukaryote mRNAs (with the exception of replication dependent histone mRNA); (ii) genes with only one exon can be targeted whereas they are not eligible for other strategies such as exon skipping; (iii) in diseases characterized by the utilization of inadequate APAs, these APAs can be targeted to promote the use of the canonical PAS. This strategy thus presents an important clinical therapeutic potential not only for muscle diseases, but also for other genetic diseases such as cancer.

## Figures and Tables

**Table 1:** Polyadenylation signals frequencies in humans and mice.

Hexameric Sequences	Human [2]	Human [4]	Mouse [4]
AAUAAA	58.2	53.18	59.16
AUUAAA	14.9	16.78	16.11
UAUAAA	3.2	4.37	3.79
AGUAAA	2.7	3.72	3.28
AAGAAA	1.1	2.99	2.15
AAUAUA	1.7	2.13	1.71
AAUACA	1.2	2.03	1.65
CAUAAA	1.3	1.92	1.80
GAUAAA	1.3	1.75	1.16
AAUGAA	0.8	1.56	0.90
UUUAAA	1.2	1.20	1.08
ACUAAA	0.6	0.93	0.64
AAUAGA	0.7	0.60	0.36
AAAAAG	0.8	-	-
AAAACA	0.5	-	-
GGGGCU	0.3	-	-
